# Automated Image-Based Fluorescence Screening of Mitochondrial Membrane Potential in *Daphnia magna*: An Advanced Ecotoxicological Testing Tool

**DOI:** 10.1021/acs.est.4c02897

**Published:** 2024-08-27

**Authors:** Cedric Abele, Amira Perez, Andrey Höglund, Paula Pierozan, Magnus Breitholtz, Oskar Karlsson

**Affiliations:** † Science for Life Laboratory, Department of Environmental Sciences, Stockholm University, 11418 Stockholm, Sweden; ‡ Stockholm University Center for Circular and Sustainable Systems (SUCCeSS), Stockholm University, 10691 Stockholm, Sweden; § Department of Environmental Science, Stockholm University, 11418 Stockholm, Sweden

**Keywords:** high-content imaging, high-content screening, JC-1, NAMs, carbonyl cyanide 3-chlorophenylhydrazone, 2,4-dinitrophenol, triclosan, 6PPD, ibuprofen, pharmaceuticals, ecotoxicology

## Abstract

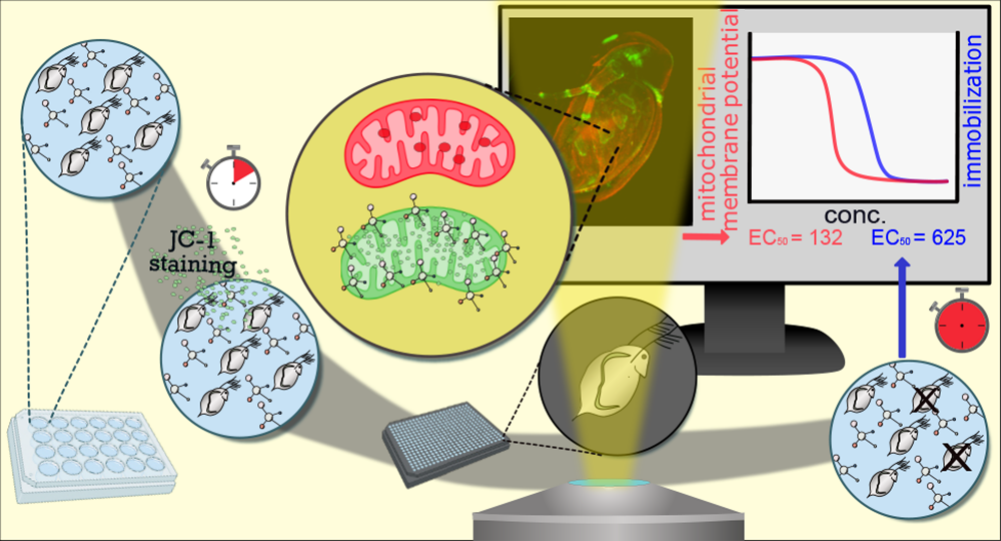

This study demonstrated the strengths of in vivo molecular staining coupled with automated imaging analysis in *Daphnia magna*. A multiwell plate protocol was developed to assess mitochondrial membrane potential using the JC-1 dye. The suitability of five common anesthetics was initially tested, and 5% ethanol performed best in terms of anesthetic effects and healthy recovery. The staining conditions were optimized to 30 min staining with 2 μM JC-1 for best J-aggregate formation. The protocol was validated with the model compound carbonyl cyanide 3-chlorophenylhydrazone (CCCP) and used to measure the effect of four environmental contaminants, 2,4-dinitrophenol, triclosan, *n*-(1,3-dimethylbutyl)-*N*′-phenyl-p-phenylenediamine (6PPD), and ibuprofen, on mitochondrial health. Test organisms were imaged using an automated confocal microscope, and fluorescence intensities were automatically quantified. The effect concentrations for CCCP were lower by a factor of 30 compared with the traditional OECD 202 acute toxicity test. Mitochondrial effects were also detected at lower concentrations for all tested environmental contaminants compared to the OCED 202 test. For 2,4-dinitrophenol, mitochondria effects were detectable after 2 h exposure to environmentally relevant concentrations and predicted organism death was observed after 24 h. The high sensitivity and time efficiency of this novel automated imaging method make it a valuable tool for advancing ecotoxicological testing.

## Abstract 2

Chemical safety assessment requires new approach methodologies to understand toxic mechanisms of chemicals, and this study demonstrates that automated in vivo imaging in *D. magna* can be a valuable tool.

## 1 Introduction

More than 2 billion tons of synthetic chemicals are produced globally each year, and new compounds are constantly developed.^1^ The resulting environmental contamination is estimated to be responsible for millions of human deaths per year and threatening tens of thousands of species to extinction.^2^ Only a tenth of the chemicals that are produced in a large scale are sufficiently tested to estimate their hazardous potential for the environment.^3^ The current standard methods for regulatory (eco)toxicity testing of chemicals are based on single species experiments mostly focusing on acute toxicity and apical end points, such as mortality and number of offspring.^4−8^ Furthermore, traditional animal testing is criticized for being slow, expensive, and insensitive and may raise ethical concerns. To change chemical production toward a safer and more sustainable future, regulatory risk assessment of chemicals requires new approach methodologies (NAMs) that provide high-quality toxicological data in a time and cost-efficient way.^2^ This includes the development of test systems that are capable of elucidating potential toxicological mechanisms underlying a chemical’s adverse effect. Such information is central for the development of adverse outcome pathways (AOPs) and allows easier extrapolation between species.^9^ Many NAMs make use of latest technologies to enable the generation of mechanistic data and also consider the 3R (replacement, reduction, and refinement) principles for animal testing by implementing molecular in vitro data and computational modeling. Nevertheless, an organism is a much more complex biological system, and understanding single isolated toxicological pathways does not always reflect the physiological processes of the organism in vivo, which makes it challenging to fully replace animal studies.^2^ Most invertebrate species do not fall under animal welfare concern according to the EU law, and methods based on *Daphnia magna* as a model organism in ecotoxicity testing are therefore not in conflict with the 3R principles.^10^

*D. magna* is a frequently applied standard test species and implemented in the OECD guidelines for testing of chemicals.^4,5,11^*D. magna* is a species of the globally distributed invertebrate superorder, Cladocera. They feed on microalgae and provide an important food source for fish, thus acting as an essential link between primary producers and secondary consumers in the food chain. They are easy to culture, have a high reproduction rate, and a short generation time. In addition, their parthenogenetic life cycle makes them particularly useful for biological testing.^12^ Since *Daphnia* are transparent, they are also suitable for image-based methods. Despite this, the OECD standard test protocols are limited to the immobilization end point (acute toxicity) and the reproduction end point (chronic toxicity) by counting the number of offspring.^4,5^

Image-based high-content screening (HCS) includes the use of automated microscopic image acquisition combined with quantitative evaluation of multiparametric data sets. The development of various fluorescent dyes and antibodies allows molecular staining and visualization of several biochemical processes.^13,100^ Due to the development of more powerful computer and imaging technology, HCS has found increasing application in cell biological studies in a high-throughput format. This noninvasive method opens new windows to observe intracellular processes and enables multicompartment data acquisition on a relatively fast scale.^14^ HCS is a well-established technique in drug discovery, particularly in pharmacological in vitro studies. However, only a few studies have conducted fluorescent staining in *D. magna* and never in an HCS manner.^15−19^ For comprehensive mechanistic understanding of a chemical’s mode of action (MoA), the test organism’s integrity and molecular stains, which enables the visualization of multiple toxicological end points, are highly relevant. The staining can serve as a sensitive and quantifiable marker for changes in biochemical processes. In addition, performing assays in a more high-throughput multiwell plate format can provide a time-efficient tool for the toxicity screening of chemicals.

Dinoseb (6-sec-butyl-2,4-dinitrophenol) and DNOC (dinitro-ortho-cresol) are two pesticides from the dinitrophenol family that act by uncoupling the oxidative phosphorylation in mitochondria.^20^ At the inner mitochondrial membrane, ATP is produced through oxidative phosphorylation, and an uncoupling effect can therefore be fatal for the cell. During the oxidative phosphorylation, a proton gradient is built up along the inner mitochondrial membrane. This mitochondrial membrane potential is the driving force that transports molecules between the mitochondrial intermembrane space and the mitochondrial matrix. It is therefore important for ionic homeostasis and metabolic processes.^21,22^ Hence, the mitochondrial membrane potential is an essential indicator of cellular and an organismal health. Since mitochondria are vital for all eukaryotic cells, dinitrophenolic compounds may be a threat to nontarget organisms, especially in water bodies nearby application sites.^20,23,24^ Although Dinoseb and DNOC have been banned in the EU and the US for many decades, 2,4-dinitrophenol is still a precursor for many industrial chemicals, and several thousand tons are produced annually.^25^ It can be found in various products and used in, for example, polymer industry, dye production, and as wood preservatives. Dinitrophenolic compounds can therefore enter the environment through several pathways and are still found in many surface waters.^25−30^ Several other classes of environmental contaminants also have the potential to directly, or indirectly, induce adverse effects on mitochondrial health in *D. magna*.

The molecular stain 5,5′,6,6′-tetrachloro-1,1′,3,3′-tetraethylbenzimidazolylcarbocyanine iodide (JC-1) can be used to study alterations in the mitochondrial membrane potential. JC-1 is a lipophilic cation that permeates through the cell and mitochondrial membranes and forms aggregates when present at high concentrations. JC-1 accumulates when the mitochondrial membrane potential is high and forms red-orange light-emitting “J-aggregates.” If the mitochondrial membrane potential is low, JC-1 stays in its green-light-emitting monomeric form. The red to green ratio is therefore a valuable measurement for mitochondrial health when JC-1 is applied.^31−35^ The main aim of this study was to demonstrate the applicability of HCS in *D. magna* by developing a JC-1 imaging protocol. The protocol was first validated using the known mitochondrial toxicant carbonyl cyanide 3-chlorophenylhydrazone (CCCP) as the model compound and thereafter applied to characterize effects of the environmental contaminants 2,4-dinitrophenol, triclosan, *N*-(1,3-dimethylbutyl)-*N*′-phenyl-p-phenylenediamine (6PPD), and ibuprofen on mitochondrial health in *D. magna*.^32,34−37^ The OECD test guideline 202 on “*Daphnia* sp., acute immobilization test”^5^ was also conducted and compared to the developed fluorescence quantification method.

## 2 Materials and Methods

### 2.1 Chemicals

JC-1, 4′,6-diamidino-2-phenylindole dihydrochloride (DAPI), CCCP (≥98% purity, CAS Number: 555-60-2), and triclosan (≥99% purity, CAS number: 3380-34-5) were obtained from Thermo Fisher (Kandel, Germany). 2,4-Dinitrophenol (≥98% purity, CAS number: 51-28-5), ibuprofen (≥98% purity, CAS number: 15687-27-1), and ethyl-3-aminobenzoate-methanesulfonate (tricaine/MS-222, ≥98% purity, CAS number: 886-86-2) were obtained from Sigma-Aldrich (Steinheim, Germany). 6PPD (≥98% purity, CAS number: 793-24-8) was obtained from TCI Europe (Zwijndrecht, Belgium). Ethanol (≥99.9% purity) and methanol (≥99.9% purity) were obtained from VWR (Fontenay-sous-bois, France) and isopropanol (≥99.8% purity) from Fisher Scientific (Geel, Belgium). Carbonated water was purchased from a common supermarket.

### 2.2 Test Organism

The “*D. magna* environmental pollution test strain clone 5” of the Federal Environmental Agency, Berlin, Germany, was cultured in glass beakers with 25 females per 2 L OECD standard medium M7.^5^ Three times a week, *D. magna* were fed with solutions of the monocellular freshwater microalgae *Raphidocelis subcapitata* (6.8 mL of ∼3.5 × 10^5^ cells/mL) and *Desmodesmus subspicatus* (1 mL of ∼3.5 × 10^5^ cells/mL). For all experiments, *D. magna* juveniles (<24 h old) from mothers older than 2 weeks were used. Prior to the experiments, the mothers were transferred to fresh M7 so that juveniles used in experiments were born in clean M7 medium.

### 2.3 Optimization of Anesthesia Protocol

Imaging of *D. magna* requires immobile individuals. Initially, it was therefore necessary to optimize anesthesia conditions. The suitability of the following anesthetics was evaluated: tricaine (or MS-222), carbonated water, methanol, ethanol, and isopropanol. Tricaine was tested at 0.5, 1.0, 1.5, and 2.0 mg/mL, carbonated water at 1 and 10%, and methanol, ethanol, and isopropanol at 1, 2, 3, and 5% in M7. Ten juveniles were exposed to the respective anesthesia solution in glass beakers with a volume of 20 mL. The time until all of the individuals were immobile was measured. If they were still moving after 15 min, it was considered as an insufficient anesthetic effect. Since images are usually taken within 0.5 and 2 h after anesthesia, the individuals were kept for another 2 h in the solutions before they were transferred to the clean M7 medium. The recovery was tested by evaluating their immobilization after 1, 24, and 48 h in the clean M7 medium.

### 2.4 Optimization of JC-1 Molecular Staining Concentration and Time

The molecular stain JC-1 and its red to green fluorescence ratio are commonly used to study mitochondrial health in cell studies. Previous studies have applied JC-1 to qualitatively study the change in the mitochondrial membrane potential in *D. magna*.^15,38^ However, this study is, to our knowledge, the first to quantitatively assess the in vivo toxicity in *D. magna* based on automated fluorescence imaging data in multiwell format. To optimize the JC-1 staining protocol in *D. magna*, different staining concentrations and staining times were evaluated.

Staining concentrations of 0.5, 1, 2, and 5 μM dissolved in M7 were tested using a staining period of 60 min according to the supplier’s manual and Teplova et al.^15^ Ten juveniles were stained in 24-well tissue culture plates (VWR, Radnor, PA, USA) using a 2 mL staining volume. The individuals were then anesthetized with 5% ethanol and transferred in a volume of 20 μL by a pipet to 384 well plates (low-volume, round, Corning, Kennebunk, ME, USA). The plates were centrifuged at 78*g* for 2 min to bring the individuals in each well to the plate bottom, and the images were acquired as described below. The optimal staining concentration was selected based on the image quality, considering distribution of the dye in the individuals and formation of J-aggregates. For a better assessment of the JC-1 staining, cell nuclei of the *D. magna* juveniles were simultaneously stained with DAPI at a final concentration of 9 μM. After the optimal staining concentration was defined (i.e., 2 μM), different staining times (15, 30, 60, and 120 min) were tested. Ten juveniles were exposed to 2 μM in 2 mL of JC-1 staining solution. After the respective staining time, half of the staining solution was removed and replaced with 10% ethanol for anesthesia to a final ethanol concentration of 5%. Anesthetized *D. magna* individuals were transferred in a volume of 20 μL by a pipet to 384 well plates (one individual per well) and centrifuged at 78*g* for 2 min before imaging.

### 2.5 Exposure to CCCP, 2,4-Dinitrophenol, Triclosan, 6PPD, and Ibuprofen in 24-Well Plates and JC-1 Staining at 2 μM

The test compound concentrations were chosen based on the results from previous immobilization experiments in glass beakers in accordance with OECD test guideline 202 and adjusted according to the hypothesis that the developed method is more sensitive (SI5). CCCP, a well-known uncoupler of the oxidative phosphorylation and often used as a positive control for assessment of adverse effects on mitochondrial health, was used as a model compound.^32,35,36^ CCCP was tested at 0.5, 5, 25, 50, 250, 500, and 2500 μg/L dissolved in M7 from a 10 g/L stock solution in DMSO. The control was exposed to M7 medium only and the solvent control to 0.025% DMSO in M7 which corresponds to the highest DMSO concentration in the CCCP experiments. Afterward, the environmental contaminants 2,4-dinitrophenol (0.94, 1.88, 3.75, 7.5, 15, 20, 30, and 40 mg/L), triclosan (0.05, 0.1, 0.2, 0.4, 0.8, 1.6, and 3.2 mg/L), 6PPD (0.06, 0.13, 0.25, 0.5, 1, 2, and 4 mg/L), and ibuprofen (1.56, 3.25, 6.25, 12.5, 25, 50, and 100 mg/L) were tested. From stock solutions in DMSO, the exposure concentrations were prepared in M7. The control was exposed to M7 medium only, and the solvent control was exposed to DMSO in M7 which corresponded to the highest DMSO concentration used in each exposure experiment (2,4-dinitrophenol: 0.02%, triclosan: 0.08%, 6PPD: 0.1%, and ibuprofen: 0.1%). The experiments were conducted in 24-well plates and each concentration was tested in one well with five individuals and an exposure volume of 2 mL. The individuals were exposed for 2 h and 24 h in parallel experiments. All experiments were performed in independent triplicates. At the end of the exposure, immobilization was measured, and then half of the exposure solution was removed and replaced with the staining solution for a final JC-1 concentration of 2 μM. After 30 min, half of the staining solution was removed and replaced with 10% ethanol for anesthesia at 5% ethanol. Immobile *Daphnia* were then transferred in a volume of 20 μL into 384 well plates with one individual per well. The multiwell plate was centrifuged at 78*g* for 2 min, and images were acquired as described below.

### 2.6 Confocal High-Content Imaging

Images were acquired with an ImageXPress Micro confocal high-content imaging system (Molecular Devices, San Jose, CA, USA). The imaging was conducted in multipoint confocal mode with a 60 μm pinhole spinning disk and a 4× objective (Plan Apo Lambda, Air, 0.2 NA, 20 mm WD, Nikon). To cover large parts of the *Daphnia* in *z*-dimension, *z*-stacks with 10 images on different *z*-levels with a distance of 15 μm were acquired. Per *z*-level, we acquired a transmitted light image and fluorescence images with the Cy3 filter set (Ex.: 531/40 nm, Em.: 593/40 nm) and the FITC filter set (Ex.: 475/34 nm, Em.: 536/40 nm). During the protocol optimization, the autofocus function in the MetaXPress software (Molecular Devices, San Jose, CA, USA) was used to find the best exposure time and adapted if necessary. The exposure times varied due to the staining concentrations and objective in the optimization experiments (SI2). In the model compound experiments, the optimized protocol (30 min staining with 2 μM JC-1) was used and fluorescence images collected at an exposure time of 200 ms in both channels, Cy3 and FITC. For more detailed observation of mitochondrial staining with JC-1, more z-levels were used, and *D. magna* were additionally imaged with a 20× objective (Plan Apo Lambda S, Water immersion, 0.95 NA, 0.99–0.90 mm WD, Nikon).

### 2.7 Image Analysis and Statistics

**Figure 1:**
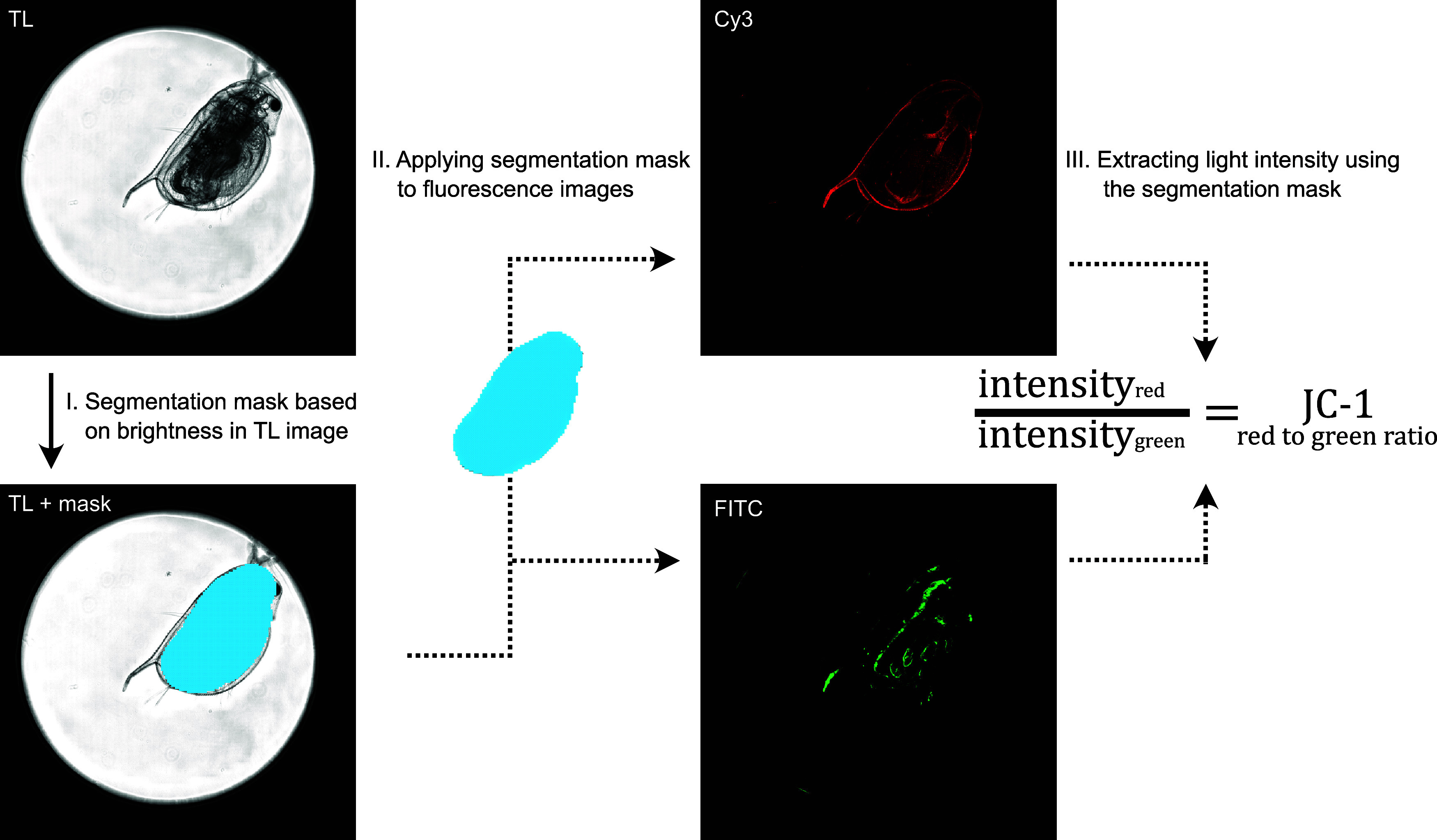
Image analysis: creation of a segmentation mask was based on the difference in brightness in the transmitted light (TL) image of *D. magna*. This mask was applied to the corresponding fluorescence images (Cy3, FITC) to quantify fluorescence intensities of JC-1 staining.

An image analysis workflow was established by using the MetaXpress Custom Module software (Molecular Devices, San Jose, CA, USA). To identify the *D. magna* outline, a segmentation mask was generated based on the transmitted light image. This mask was used to measure the fluorescence intensity of the corresponding Cy3 and FITC images and also to normalize the JC-1 signal by the area of *Daphnia* (Figure 1).

The image analysis workflow measures the number of segments found per image, average Cy3 intensities, and average FITC intensity in the segmentation mask. Overexposed parts of the image were excluded from the analysis by thresholding the intensity in the Cy3 channel at 65000. The results of the image analysis were saved as an excel output file containing the information mentioned above in separate sheets. The .xlsx-file was then processed with a custom R script to exclude wells with erroneous segmentation. Since one *D. magna* was placed per well, only wells with a segmentation of 1 were considered as correct. A segmentation number of 0 means that the software was unable to find the outline of the *D. magna*. The number of segments higher than 1 indicates that additional *Daphnia* unintentionally were added per well or incorrect segmentation because of artifacts in the images. If more than 50% of *z*-layers per image were incorrect, the entire individual was excluded. For quantification of mitochondrial health, the R script calculated the red to green fluorescence ratio in every image. The red to green ratio per individual was calculated as the average of the *z*-levels.

The average red to green ratios of five replicates per concentration from all three independent experiments were used to fit a concentration–response model using the drc package and to calculate EC_10_s and EC_50_s for each compound. The immobilization data were modeled with the two-parameter log–logistic function with upper and lower limits fixed to 0 and 1, respectively. For the JC-1 intensity data, all intensities were normalized to the M7 medium control, and a four-parameter log–logistic function was applied, while the lower limit was fixed to the average intensity of dead controls.^39,40^ The concentration–response data were presented as mean for each experimental group consisting of five individuals, the concentration–response curve, and the 95% confidence intervals. The data were checked for normality, and the M7 medium control and DMSO control were tested for significant differences. All R-scripts can be found on github (https://github.com/flerpan01/imageXPress_plotter).

### 2.8 ATP Analysis

The level of ATP in *D. magna* exposed to 250 or 2500 μg/L CCCP for 2 h was measured using a commercial kit (ATP assay kit/ab83355, Abcam, Cambridge, U.K.) according to the instructions. Ten *D. magna* were treated per group, and four independent experiments were conducted. The ATP assay protocol relies on the phosphorylation of glycerol to generate a product that is quantified by fluorescence (Ex/Em = 535/587 nm). The results are shown as mean ± SD and differences compared to the control analyzed by one-way analysis of variance (ANOVA), followed by Dunnet’s multiple test.

## 3 Results

### 3.1 Anesthesia

Since imaging requires immobile individuals for up to 2 h, five anesthetics commonly used on aquatic organisms were tested in *D. magna* at different concentrations. The time until all individuals were immobile was measured. The results demonstrated that the juveniles were immobile within 15 min at tricaine concentrations of ≥1 mg/mL and 10% of carbonated water (Table 1). However, when the juveniles were transferred back to the clean M7 medium, they did not recover. *D. magna* were successfully anesthetized within 5 min with 5% methanol, and all individuals fully recovered when transferred back to the clean M7 medium after 2 h exposure. Isopropanol and ethanol concentrations ≥2% also induced successful immobilization within 5 min. The individuals recovered fully from isopropanol exposure at 2% and 3%, but only 2 out of 10 organisms recovered after anesthesia with 5% isopropanol. Full recovery was achieved after anesthesia with ethanol at all tested concentrations, and 5% ethanol was used for all imaging experiments.

**Table 1:** Percentage of *D. magna* Recovered after 2 h Immobile in Anesthesia Solution^a^

anesthetic	time until immobilization	recovery		
		1 h	24 h	48 h
control	–	100%	100%	100%
tricaine (0.5 mg/mL)	–	–	–	–
tricaine (1.0 mg/mL)	<15 min	0%	0%	0%
tricaine (1.5 mg/mL)	<15 min	0%	0%	0%
tricaine (2 mg/mL)	<15 min	0%	0%	0%
carbonated water (1%)	–	–	–	–
carbonated water (10%)	<10 min	0%	0%	0%
methanol (1%)	–	–	–	–
methanol (2%)	–	–	–	–
methanol (3%)	–	–	–	–
methanol (5%)	<1 min	100%	100%	100%
isopropanol (1%)	–	–	–	–
isopropanol (2%)	<5 min	100%	100%	100%
isopropanol (3%)	<1 min	100%	100%	100%
isopropanol (5%)	<1 min	20%	50%	20%
ethanol (1%)	–	–	–	–
ethanol (2%)	<1 min	100%	100%	100%
ethanol (3%)	<1 min	100%	100%	100%
ethanol (5%)	<1 min	100%	100%	100%

a Concentration which did not lead to sufficient anesthesia are marked with “–”.

### 3.2 JC-1 Staining Concentration

**Figure 2:**
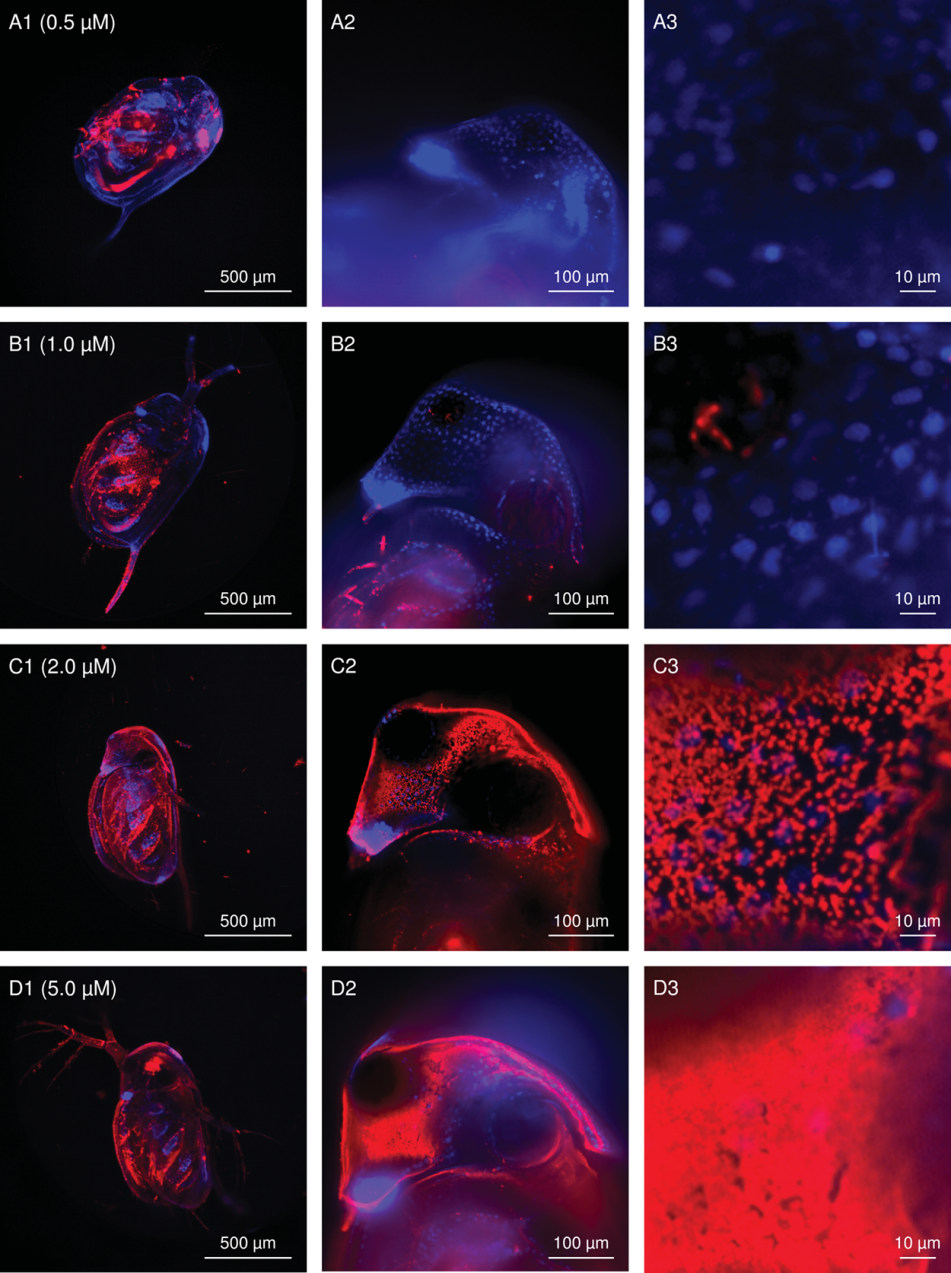
Images show an overlay of nuclei staining with DAPI (blue) and the JC-1 signal in the Cy3 channel (red) after staining *D. magna* with 0.5, 1.0, 2.0, or 5.0 μM for 30 min. Column I (A1, B1, C1, and D1) was acquired with a 4× air objective and shows the 2D projection of 50–100 *z*-stacks. Images in column II (A2, B2, C2, and D2) were acquired with a 20× water immersion objective focusing on *D. magna* head. A representative *z*-layer was selected. Column III (A3, B3, C3, and D3) is a digital zoom of the images in column II.

For quantification of the fluorescent intensity, the staining concentration and staining time needed to be optimized for *D. magna* juveniles. Four different concentrations were tested and optically validated. Figure 2 shows the distribution of JC-1 in *D. magna* at 0.5, 1.0, 2.0, and 5.0 μM JC-1 staining and cell nuclei stained with DAPI. J-aggregates were detected at all tested JC-1 concentrations, and their abundance increased with increasing staining concentration. At staining concentrations of 0.5 μM and 1.0 μM, the dye was detected mainly in the region of their thoracic appendages (Figure 2A1,B1), while no dye was able to enter the head region (Figure 2A2,B2). JC-1 concentrations of 2.0 μM and 5.0 μM resulted in an even distribution in the *Daphnia* body (Figure 2C1,D1) and staining of the head region (Figure 2C2,D2). At 2 μM, JC-1 J-aggregates with a size of 0.5 to 1 μm surrounding the cell nuclei could be observed (Figure 2C3). At 5 μM JC-1, an accumulation was observed in the head region, and it was difficult to distinguish individual J-aggregates (Figure 2D3) due to patches of high intensities in *D. magna* (Figure 2D1–D3). Agglomeration of the JC-1 dye outside of the organism increased with higher JC-1 concentrations (SI3). Based on these studies, 2 μM JC-1 was selected as the optimal concentration.

### 3.3 JC-1 Staining Time

After finding the optimal JC-1 concentration, *D. magna* individuals were stained with 2 μM JC-1 at four different incubation times. Our data show that the red to green fluorescence ratio of the JC-1 staining reached the plateau after 15 min, and no significant differences were detected between a staining time of 15 min and up to 120 min (SI4, *p* > 0.05).

### 3.4 Effects of the Model Compound CCCP

**Figure 3:**
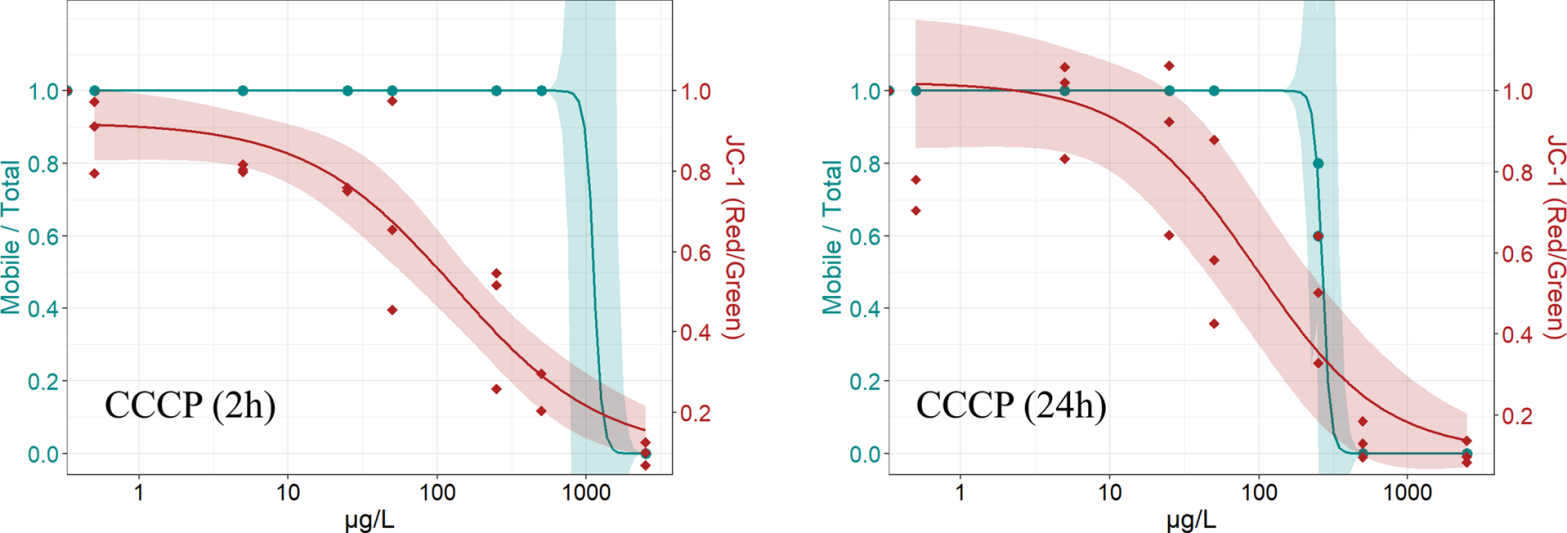
Graphs show the immobilization of *D. magna* (blue) and corresponding red/green ratio of the JC-1 signal (red) from the automated image-based screening of the mitochondrial membrane potential after 2 and 24 h of exposure to carbonyl cyanide 3-chlorophenylhydrazone (CCCP). The data are derived from three independent experiments, with each individual data point representing the average of five replicates. The corresponding concentration–response model and 95% confidence intervals are shown.

The effect of CCCP exposure on the *D. magna* mitochondrial membrane potential and immobilization was tested at a concentration range of 0.5 to 2500 μg/L in 24-well plates using the optimized JC-1 protocol. There was no significant difference in effect concentrations for immobilization between the developed 24-well plate protocol and the OECD *Daphnia Sp*. Acute Immobilization Test setup. The test concentration was adapted due to the effects on the mitochondrial membrane potential at much lower CCCP concentrations compared to effects on the immobilization, leading to large confidence intervals in the immobilization data in the 24-well plate setup due to missing data between 500 and 2500 μg/L. Furthermore, no significant difference in the JC-1 signal between the M7 control and DMSO control was shown. Exposure to CCCP for 2 h led to 100% immobilization at 2500 μg/L, while no effect was observed at concentrations of up to 500 μg/L (Figure 3). Compared to the immobilization test, the EC_50_ of the JC-1 signal was lower by a factor of 5 after 2 h of exposure. EC_10_ of the JC-1 signal is by a factor of 30 lower than the EC_10_ of immobilization after 2 h (Table 2). After 24 h exposure to CCCP, effects in the mobility were detected at 250 μg/L, and more than 90% of the individuals are immobile at 500 μg/L.

**Table 2:** EC_50_ and EC_10_ with Corresponding 95% Confidence Intervals for Immobilization in the OECD Setup and JC-1 Signal from the Image-Based Screening of Mitochondrial Membrane Potential in *D. magna* after 2 and 24 h Exposure to CCCP in 24-Well Plates

end point	2 h (μg/L)	24 h (μg/L)
EC_50_ (immobilization, OECD protocol)^a^	625.2 (484.8–765.1)	291.1 (222.6–269.5)
EC_50_ (immobilization, 24-well plate protocol)	1120.4 (−11007 to 13248.4)	266.2 (147.5–385.0)
EC_50_ (JC-1 red/green, 24-well plate protocol)	132.1 (47.2–217.1)	95.2 (7.0–183.5)
EC_10_ (immobilization, OECD protocol)^a^	327.3 (212.6–442.1)	133.5 (79.2–187.8)
EC_10_ (immobilization, 24-well plate protocol)	970.0 (−9748 to 11688)	169.9 (99.5–240.3)
EC_10_ (JC-1 red/green, 24-well plate protocol)	10.9 (−6.7 to 28.6)	11.0 (−9.6 to 31.5)

a Supporting Information (SI5).

The effect concentrations for the JC-1 signal were also lower after 24 h exposure but did not change significantly compared to 2 h exposure, whereas the effect concentrations of the immobilization decreased by a factor of ∼2.1 (Table 2). The effect concentrations of JC-1 signals were significantly lower than for the immobilization data (no overlap of 95% confidence interval).

### 3.5 Effects of CCCP on ATP Levels

**Figure 4:**
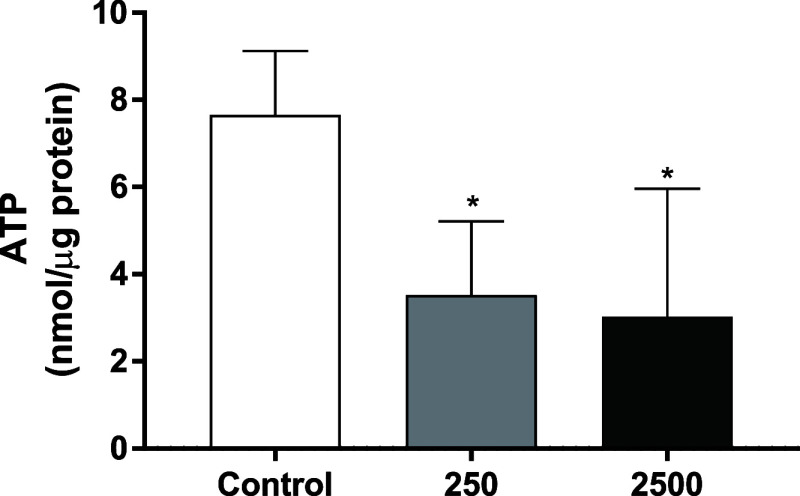
Effect on ATP levels after 2 h of exposure to 250 and 2500 μg/L CCCP. Values represent mean ± SD from four independent experiments with ten individuals for each concentration. Statistically significant differences from control are indicated as follows: *p < 0.05 (ANOVA followed by Dunnet’s multiple comparison test).

To validate that the decrease in the JC-1 signal caused by CCCP reflects an effect on mitochondria, we measured the ATP levels in *D. magna* treated with 250 or 2500 μg/L for 2 h. The results showed that CCCP caused a decrease in the ATP levels in both concentrations (Figure 4), confirming that the decreased signal of JC-1 is caused by an alteration in the mitochondria function.

### 3.6 Effects of Environmental Contaminants

*D. magna* were exposed to 2,4-dinitrophenol, triclosan, 6PPD, and ibuprofen in relevant concentration ranges between no effect and immobilization to examine effects on the mitochondrial membrane potential and investigate the sensitivity of the automated JC-1 imaging method. In accordance with the CCCP experiments, no significant difference between the immobilization data from the 24-well plate protocol and the data from the OECD guideline protocol was observed for 2,4-dinitrophenol, triclosan, and ibuprofen (Tables 3 and SI5). The EC_10_ of 6PPD in glass beakers was lower than that in 24-well plates. There was no significant difference in the JC-1 signal between the M7 control and DMSO control in any of the experiments.

**Table 3:** EC_50_ and EC_10_ with corresponding 95% confidence intervals for *D. magna* Immobilization in 24-Well Plates and JC-1 Signal from the Image-Based Screening of Mitochondrial Membrane Potential after 2 and 24 h Exposure to 2,4-Dinitrophenol, Triclosan, *N*-(1,3-dimethylbutyl)-*N*′-phenyl-p-phenylenediamine (6PPD), and Ibuprofen^a^

chemical	EC_*x*_	end point	2 h (mg/L)	24 h (mg/L)
**2,4-dinitrophenol**	**EC**_**50**_	immobilization	35.4 (32.4–38.4)	15.9 (14.2–17.5)
		JC-1	21.6 (16.6- 26.6)	7.4 (5.3–9.6)
	**EC**_**10**_	immobilization	29.2 (25.2–33.3)	12.2 (9.7–14.8)
		JC-1	11.4 (5.1–17.8)	3.7 (1.1- 6.2)
**triclosan**	**EC**_**50**_	immobilization	1.64 (1.28–1.99)	0.75 (0.57–0.94)
		JC-1	1.75 (1.25–2.27)	0.20 (0.07–0.32)
	**EC**_**10**_	immobilization	1.43 (0.11–2.75)	0.35 (0.21–0.49)
		JC-1	1.22 (0.12–2.32)	0.02 (0.00–0.04)
**6PPD**	**EC**_**50**_	immobilization	3.48 (0.06–6.90)	2.22 (0.44–4.00)
		JC-1	5.58 (−0.15–11.31)	1.01 (0.20–1.84)
	**EC**_**10**_	immobilization	3.09 (−2.47–8.65)	1.95 (1.56–2.35)
		JC-1	0.64 (−0.65–1.84)	0.09 (−0.08–0.25)
**ibuprofen**	**EC**_**50**_	immobilization	no effect	95.23 (65.20–125.27)
		JC-1	119.86 (52.26–187.46)	110.46 (22.43–198.50)
	**EC**_**10**_	immobilization	no effect	62.58 (−21.32–184.80)
		JC-1	40 (−2.47–8.65)	50.93 (−27.82–129.68)

a The data are derived from three independent experiments, with each individual data point representing the average of five replicates.

The automated image-based JC-1 screening of the mitochondrial membrane potential showed significantly lower effect concentrations compared to the immobilization test for 2,4-dinitrophenol and ibuprofen after 2 h and for 2,4-dinitrophenol, triclosan, and 6PPD after 24 h (no overlap of 95% confidence interval, Table 3).

**Figure 5:**
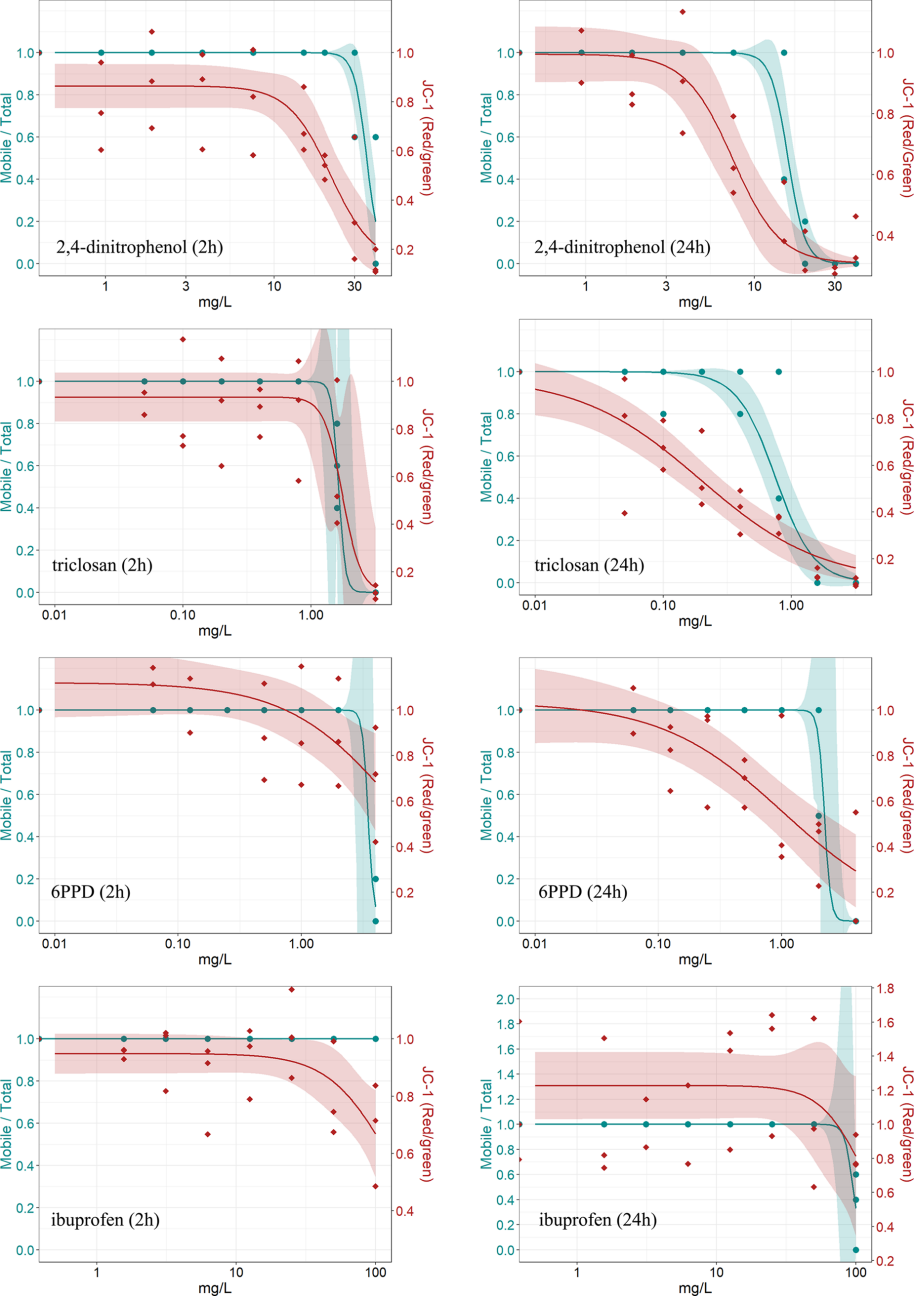
Graphs show the immobilization of *D. magna* (blue) and corresponding red/green ratio of the JC-1 signal (red) from the automated image-based screening of the mitochondrial membrane potential after 2 and 24 h of exposure to 2,4-dinitrophenol, triclosan, *N*-(1,3-dimethylbutyl)-*N*′-phenyl-p-phenylenediamine (6PPD), and ibuprofen. The data are derived from three independent experiments, with each individual data point representing the average of five replicates. The corresponding concentration–response model and 95% confidence intervals are shown.

After 2 h exposure to ≥30 mg/L 2,4-dinitrophenol, no immobilization was observed. The highest concentration (40 mg/L) resulted in 40% immobilization but only in one out of three experiments. In contrast, the JC-1 imaging revealed that 2 h 2,4-dinitrophenol exposure induced a concentration-dependent decrease in the mitochondria membrane potential with a decreasing signal already at 20 mg/L. After 24 h exposure, a clear concentration–response relationship was observed for both the immobilization assessment and the JC-1 signal (Figure 5). Interestingly, 24 h exposure to 7.5 mg/L 2,4-dinitrophenol did not lead to immobilization in *D. magna* in any of the experiments, while the imaging method revealed a decrease in the mitochondrial membrane potential. After 2 h, the EC_50_ of the JC-1 signal was lower by a factor of ∼1.5 compared to EC_50_ of immobilization, and the EC_10_ of the JC-1 signal was lower by a factor of ∼2.6 compared to immobilization. After 24 h, the EC_50_ was lower by a factor of ∼2 and EC_10_ ∼ 3.3. Notably, there were no significant differences between the effect concentration derived from JC-1 imaging after 2 h and the corresponding effect concentration of immobilization after 24 h (Table 3).

After 2 h exposure to triclosan, 3.2 mg/L caused 100% immobilization, and the JC-1 signal followed a similar trend as the immobilization data. The effect concentrations did not differ significantly from each other (Table 3). However, after 24 h, the JC-1 signal decreased at lower concentration, and the EC_50_ of 0.20 mg/mL was significantly lower by a factor of 3.75 compared to the EC_50_ of immobilization. The exposure to 6PPD showed no significant difference between the JC-1 signal and immobilization data after 2 h. Nevertheless, after 24 h exposure, the JC-1 signal decreased at lower concentrations, and the EC_10_ is significantly lower than in the immobilization test. Ibuprofen induced no immobilization of daphnia after 2 h exposure, while the JC-1 signal decreased at 100 mg/L, resulting in an EC_50_ of 119.9 mg/L. After 24 h exposure to ibuprofen, daphnia were only immobile at 100 mg/L. At this time point, an increasing JC-1 signal was measured in two out of three experiments with increasing ibuprofen concentration, before the signal decreased at 100 mg/L compared to the control. No difference between the effect concentrations was observed at 24 h (Table 3).

## 4 Discussion

The aim of this study was to develop the first automated imaging protocol for a high-content screening method in *D. magna* using multiwell plates and molecular dye JC-1 as a marker for mitochondrial health. Initially, ethanol was shown to be the most suitable anesthetic to enable live imaging of *D. magna*. The JC-1 staining conditions were then optimized, and an image analysis workflow was established. Following exposure to the model compound CCCP, known for the disruptive effect on the mitochondrial membrane potential, the developed imaging method was demonstrated to be more sensitive than the OECD immobilization test. The CCCP-induced decrease of the JC-1 signal was cross-validated by a measured decrease in ATP levels. The imaging method was then applied to four environmental contaminants (2,4-dinitrophenol, triclosan, 6PPD, and ibuprofen), and the results revealed changes in the mitochondrial membrane potential at lower concentrations compared to the immobilization data. The method was able to predict a lethal outcome in *D. magna* after a short exposure time to CCCP and 2,4-dinitrophenol.

Since no similar studies have been reported in *D. magna*, it was necessary to first find an anesthetic compound suitable for the development of the automated imaging method. The results demonstrated that ethanol was the most suitable anesthetic agent in terms of organism immobilization and recovery. An anesthetic with properties keeping the individuals completely immobile during imaging and, at the same time, having minimal effect on their health was required to minimize the interference with the actual test compound and guarantee automated image acquisition of comparable images. The compound tricaine, which is commonly used for anesthesia of aquatic organisms, is not suitable for *D. magna* imaging because the organisms could not recover from the anesthesia. This is contrary to the findings of Bunescu et al.^57^ but in line with Gannon and Gannon.^58^

The optimization of the JC-1 staining conditions is crucial for a robust test outcome, and 2 μM JC-1 for a staining time of 30–60 min was demonstrated to result in even J-aggregate formation throughout the entire body of *D. magna*. JC-1 is a frequently used molecular dye to test the mitochondrial health in various cell models and is therefore a well-validated fluorophore. Nevertheless, only a few previous studies have applied it to *D. magna*, and our images are the first that show clear J-aggregate formation in living animals surrounding the cell nuclei.^15,19,38^ We observed J-aggregate formation in the cells of *D. magna* at staining concentrations ≥0.5 μM JC-1. A higher abundance of J-aggregates was observed with increasing staining concentrations. This means that more mitochondria can be captured, and a higher sensitivity is possible. Staining of the whole *D. magna* body with even distribution of the dye could only be achieved at JC-1 concentration ≥2 μM. JC-1 is taken up passively into the cells and their mitochondria. Concentrations <2 μM are presumably not high enough to form J-aggregates in the cells of *D. magna*.^33,59^ Since *D. magna* actively filter the water with their thoracic appendages, they may create a local increase of the JC-1 concentration in the thoracic region, leading to a higher dye uptake. *D. magna* have a bivalved shell, and dye could simply be trapped and accumulated inside their thoracic body cavity, which could explain the J-aggregate formation at concentrations <2 μM.^12^ We also detected increasing occurrence of stain agglomerates outside the *Daphnia* body with increasing staining concentration and observed patches of high intensities in animals stained with 5 μM JC-1. Here, we suspect agglomerates attached to the outside of the shell. This indicates that too high staining concentrations may lead to unspecific J-aggregate formation in the staining solution independent of the mitochondrial membrane potential in *D. magna*. J-aggregate formation outside of *D. magna* had no influence on the quantification of the JC-1 signal, as they were removed in the image analysis process. In the 5 μM JC-1 staining group, however, we observed entangling of these aggregates in antennas, leading to impaired mobility and thus an adverse effect on their viability. Unspecific J-aggregate formation can also be reduced by appropriate preparation of the staining solution. Temperature might have an influence on J-aggregate formation and JC-1 solution should therefore be thawed in time.^33^ In addition, repeated freezing/thawing cycles can influence the dye performance, and JC-1 should therefore be stored in aliquots at −20 °C.^36^ We also want to point out that although *D. magna* is transparent, the chitinous shell, parts of their inner organs, and muscles are not fully transparent. In the center of *D. magna*, their body parts become rather translucent, which causes light diffusion and leads to lower intensities. Therefore, images were only collected until reaching the body center. Moreover, centrifugation led to overall good positioning of *D. magna* and enabled comparable images between the well throughout the automated image acquisition.

Performing the experiments in multiwell plates is key for conducting high-content screening and can make ecotoxicity testing both more cost- and time-efficient. The acute toxicity of all compounds on *D. magna* was tested in both 24-well plates and glass beakers according to the OECD guidelines, and the results confirmed that the 24-well plate format did not affect the test outcome for 2,4-dinitrophenol, triclosan, and ibuprofen. Effect concentrations for 6PPD in glass beakers were slightly lower compared to experiments in well plates. The immobilization EC_50_ of 2,4-dinitrophenol (13.6 mg/L), triclosan (0.42 mg/L), 6PPD (0.93 mg/L), and ibuprofen (57.35 mg/L) were similar to previous studies.^37,41−45^ No acute toxicity data for CCCP in *D. magna* could be found in the literature, but our EC_50_ of immobilization after 48 h of 63.9 μg/L is 3 orders of magnitude lower compared to 159.11 mg/L in *Daphnia pulex*, which is provided in the safety data sheet.

The developed JC-1 imaging method detected toxic effects at lower concentrations compared with those of the traditional OECD immobilization method. The method is clearly more sensitive for compounds that act as uncouplers of oxidative phosphorylation. The decrease in the JC-1 signal and hence in mitochondrial membrane potential after 2 h exposure to CCCP and 2,4-dinitrophenol, resulted in organism immobilization at 24 h. JC-1 might therefore be a predictor of lethal effects occurring after more long-term exposure. Previous studies have shown that 24 h exposure to 1.5 mg/L 2,4-dinitrophenol reduced the fatty acid metabolism in *D. magna*, which is relevant for the energy homeostasis and directly connected to mitochondrial health.^42^ At this concentration, a decrease in the mitochondrial membrane potential (EC_10_) was observed in our study, whereas no immobilization was observed within a 48 h test duration. Effects on energy homeostasis may cause major disadvantages to *D. magna* under natural conditions and under long-term exposure and is therefore a relevant end point for ecotoxicological assessment. 2,4-Dinitrophenol concentrations up to 1.1 mg/L have been detected in wood waste leachates in Canada.^28^ Similar concentrations were also found in groundwater of agricultural areas in Nigeria, and the surface water runoff in France contained up to 1.2 mg/L 2,6-dinitrophenol and a total phenol and nitrophenol concentration of more than 6 mg/L.^26,27^ These examples of monitoring studies highlight the importance of sensitive test methods that enable the detection of adverse effects at environmentally relevant concentrations.

CCCP and 2,4-dinitrophenol have a similar MoA, but the demonstrated effects on both mitochondrial membrane potential and immobilization show that 2,4-dinitrophenol is less potent than CCCP. Both compounds act as proton carriers that transport protons from the mitochondrial intermembrane space through the inner membrane into the matrix and bypassing the ATP synthase, which lowers the mitochondrial membrane potential and inhibits the phosphorylation of ADP to ATP.^22,46^ However, studies have reported 2,4-dinitrophenol to be a less specific uncoupler of the inner mitochondrial membrane and oxidative phosphorylation in cancer cells than CCCP and that it activates numerous other receptors, leading to cytotoxicity.^46^ This may explain the narrower range between the mitochondrial effect and immobilization of *D. magna* in this study. A common way to distinguish between specific and baseline toxicity in in vitro reporter gene assays is to calculate the so-called toxic ratios by dividing the effect concentration for the calculated baseline toxicity by the measured effect concentration.^47^ Similarly, toxic ratios could be calculated from the difference between the effects of JC-1 and immobilization. If applying this on the data derived in the present study, toxic ratios >10 were achieved for CCCP which would be classified as specific toxicity toward the mitochondrial membrane potential, while 2,4-dinitrophenol would be only moderately specific.^48^ Considering that mitochondria are particularly effected by baseline toxicity, at low toxic ratios, JC-1 signals might predict cytotoxicity and consequently immobilization in *D. magna* after longer exposure times.^49^

Reduced mitochondrial health is often a consequence of other preceding toxic mechanisms. The observed decrease in the mitochondrial membrane potential after 24 h, but not after 2 h, exposure to the biocide triclosan and the rubber stabilizing agent and emerging environmental contaminant 6PPD suggests that the mitochondria are not their main target site but rather an important downstream effect. Previous studies have demonstrated the mitochondrial disruptive effect of 6PPD and its derivate 6PPD quinone and triclosan through various pathways.^50−52^ Triclosan is found in many surface waters and in high concentrations in wastewater. The mitochondrial effects were detected at lower concentrations than measured in wastewaters in the US (0.09 mg/L).^53^ Contamination from 6PPD and its transformation products results mainly from surface water runoff and reaches high peak concentrations during rainfall, melting, or storm events.^54^ The effects observed after 6PPD exposure were not detected at environmentally relevant water concentrations. Contamination of 6PPD in water is dominated by transformation products, particularly the more toxic 6PPD quinone. The mitochondrial effects observed only after 24 h 6PDD exposure could therefore be a result of the toxicity of its transformation product.^51,55^ The pharmaceutical ibuprofen can be found in many wastewater effluents and is typically detected in surface waters at concentrations below 1 μg/L,^56^ where no acute toxicity or mitochondrial effect was detected after 2 and 24 h exposure in this study. However, the mitochondrial membrane potential decreased after 2 h of exposure to 100 mg/L ibuprofen, a concentration that caused the juveniles to become immobile after 24 h.

Taken together, this study shows that 2,4-dinitrophenol affects mitochondrial health in *D. magna* at environmentally relevant concentrations (EC_10_ at 24 h: 3.7 (1.1–6.2) mg/L) and demonstrates the potential of image-based methods for increased mechanistic toxicological understanding in this standard ecotoxicological test species. With the application of additional dyes, multiplexed analysis could largely increase the toxicological data and help understand the mechanistic links by a minimal increase in time effort.

## Supporting Information Available

*D. magna* culture, microscope settings, staining concentration, staining times, and 2, 24, and 48 h acute toxicity data of CCCP, 2,4-dinitrophenol, triclosan, 6PPD and ibuprofen (PDF)
