# Correction to “Automated Image-Based Fluorescence Screening of Mitochondrial Membrane Potential in *Daphnia magna*: An Advanced Ecotoxicological Testing Tool”

**DOI:** 10.1021/acs.est.5c05506

**Published:** 2025-05-26

**Authors:** Cedric Abele, Amira Perez, Andrey Höglund, Paula Pierozan, Magnus Breitholtz, Oskar Karlsson

In Table 1, corrections being made to the original paper are listed, including a corrected version of Table SI3 in the accompanying Supporting Information.

**Table 1:** List of Corrections

location	error	correction
Table 2, row 1, column 3	291.1 (222.6–269.5)	296.1 (222.6–369.5)
Table 2, row 5, column 3	169.9 (99.5–240.3)	232.3 (110.6–353.9)
Table 3, row 12, column 4	0.64 (−0.65 to 1.84)	0.64 (**−**0.56 to 1.84)
Table 3, row 15, column 5	62.58 (−21.32 to 184.80)	81.74 (−21.32 to 184.80)
Table 3, row 16, column 4	40 (−2.47 to 8.65)	40.88 (−0.04 to 81.80)
Table SI3, CCCP, 6PPD, ibuprofen, triclosan	EC10s and EC50 values were inadvertently swapped	corrected table in the Supporting Information
Table SI3, 2,4-dinitrophenol	values were placed in the incorrect column and row	corrected table in the Supporting Information

## Supplementary Material



## Notes

Additional information about *D. magna* cultures, microscope settings, staining concentrations, staining times, and 2, 24, and 48 h acute toxicity data of CCCP, 2,4-dinitrophenol, triclosan, 6PPD, and ibuprofen

