# Supplementary material for: Automated Image-Based Fluorescence Screening of Mitochondrial Membrane Potential in Daphnia magna: An Advanced Ecotoxicological Testing Tool
Source: Environ Sci Technol. 2024 Aug 27;58(36):15926–37. doi: 10.1021/acs.est.4c02897 (PMC11393999; doi:10.1021/acs.est.4c02897)
Supplement: Supplementary file 1 — es4c02897_si_001.pdf [file es4c02897_si_001.pdf]

Automated Image-based Fluorescence Screening of Mitochondrial Membrane Potential in *Daphnia magna*: An advanced ecotoxicological testing tool

Cedric Abele[1,2], Amira Perez[1,2], Andrey Höglund[1,2], Paula Pierozan[1,2], Magnus Breitholtz[2,3] and Oskar Karlsson[1,2*]

[1]Science for Life Laboratory, Department of Environmental Sciences, Stockholm University, 114 18 Stockholm, Sweden

[2]Stockholm University Center for Circular and Sustainable Systems (SUCCeSS), Stockholm University, 106 91 Stockholm, Sweden.

[3]Department of Environmental Science, Stockholm University, 114 18 Stockholm, Sweden

*Corresponding author: Oskar Karlsson, Science for Life Laboratory, Department of Environmental Science, Stockholm University, 11418, Stockholm, Sweden..

E-mail: Oskar.Karlsson@aces.su.se

Number of pages: 8

Table of content

SI1 – Preparation of *D. magna* food algae 3

SI2 – Exposure times for different JC-1 concentrations and objectives 4

SI3 – Unspecific JC-1 agglomerates outside of the *D. magna* body 4

SI4 – Staining time 5

SI5 – OECD *D. magna* acute immobilization test for CCCP, 2,4-dinitrophenol, triclosan, 6PPD and ibuprofen 6

Tables

Table SI1. List of nutrients to prepare MBL for food algae preparation 3
Table SI2. Different exposure times depending on filter set and objectives that were used for the experiments in this study..4
Table SI3. Dose-response curve and EC10 and EC50 values with corresponding 95 % confidence intervals of D. magna after 2 h and 24 h of exposure to CCCP, 2,4-dinitrophenol, triclosan, 6PPD, ibuprofen. 7

Figures

Figure SI1. Unspecific JC-1 aggregate formation in the well outside of *D. magna* body and staining concentration of 5 µM 4
Figure SI2: Red-green-ratio of unexposed D. magna after different incubation times of JC-1 staining. Three independent experiments are shown. Error bars show standard deviation (n = 10) and no significant differences were measured between 15- and 120 min staining time (one-way ANOVA and Tuckey p > 0.05). 5

SI1 - Preparation of *D. magna* food algae

To prepare the algae growth medium add 750 mL MQ water into a 1 L volumetric flask, then add the following stock solutions:

Table SI1. List of nutrients to prepare MBL for food algae preparation

**Micro Nutrients**		
amount per 1L		concentration
1 ml	Na_2EDTA	4.36 g/L
2 ml	$FeCl_3$* 6H2O	3.15 g/L
1 mL	**trace element solution**	
	$CuSO_4$ *$5H_2O$	0.01 g/L
	$CoCl_2$*$6H_2O$	0.01 g/L
	$ZnSO_4$*$7H_2O$	0.022 g/L
	$MnCl_2$ *$4H_2O$	0.18 g/L
	Na_2MoO_4*H_2O	0.006 g/L
	H_3BO_3	1 g/L
**Macro nutrients**		
1 ml	$CaCl_2$*$2H_2O$	36.76 g/L
1 ml	$MgSO_4$*$7H_2O$	36.97 g/L
1 ml	$NaHCO_3$	12.6 g/L
1 ml	K_2HPO_4	8.71 g/L
1 ml	$NaNO_3$	85.01 g/L

Fill up to 1 L with MQ.

Add 500 mL MBL to 2 L Erlenmeyer flask with a stopper/cotton plug and cover with aluminium foil and autoclave the flask. Let cool down before use.

1. Inoculation and algae growth

a) Under sterile conditions add 500 µL Na_2SiO_3*$9H_2O$ (28.42 g/L, sterile) to 500 mL MBL.

b) Under sterile conditions inoculate the new culture by adding a few millilitres algae solution from primary culture.

c) Put the new cultures on the shaker and let grow for 6 days.

d) After 6 days add 500 µL vitamins to the algae. Let the algae grow for additional 24 h.

e) Under sterile conditions transfer 2x500 mL algae to a 1 L autoclaved Duran flask. Put the Duran flasks into the dark to let the algae sediment.

f) Once the algae have sedimented (after 5-7 days) remove the MBL using the water pump jet. Resuspend the algae in M7:

3L *R. subcapitata* (MBL) = 200 mL food algae (M7).

1L *D. subspicatus* (MBL) = 100 mL food algae (M7)

SI2 – Exposure times for different JC-1 concentrations and objectives

Table SI2. Different exposure times depending on filter set and objectives that were used for the experiments in this study

Experiment	Cy3		FITC		DAPI	
Objective	4x	20x	4x	20x	4x	20x
JC-1 (0.5 µM)	1000 ms	500 ms	1000 ms	500 ms	5000 ms	1000 ms
JC-1 (1.0 µM)	350 ms	1000 ms	350 ms	1000 ms	1500 ms	200 ms
JC-1 (2.0 µM)	200 ms	130 ms	200 ms	130 ms	5000 ms	1000 ms
JC-1 (5.0 µM)	100 ms	200 ms	100 ms	200 ms	5000 ms	5000 ms

SI3 – Unspecific JC-1 agglomerates outside of the D. magna body

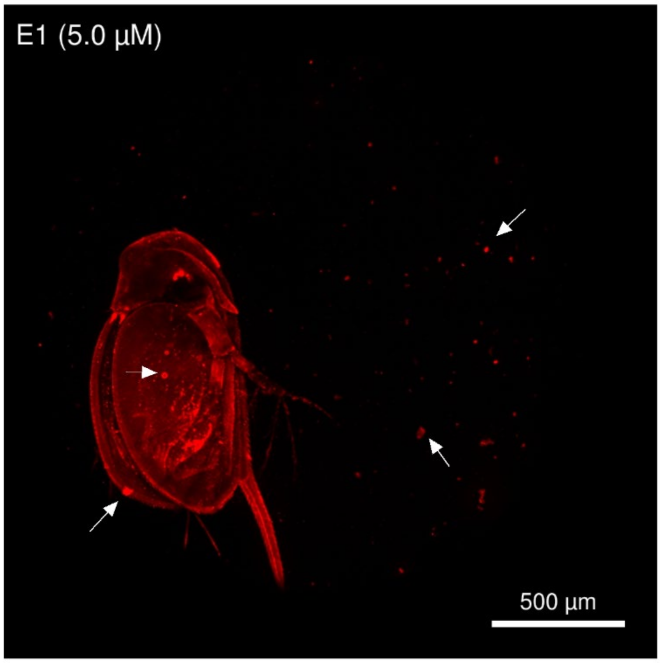

Figure SI1. Unspecific JC-1 aggregate formation in the well outside of D. magna body and staining concentration of 5µM

SI4 – Staining time

For the optimisation of the staining time, the results are presented as mean with standard deviation for each experimental group with ten individuals. One-way ANOVA followed by the Tukey post doc test was used to identify significant differences between the staining times. No significant difference between the staining times could be measured.

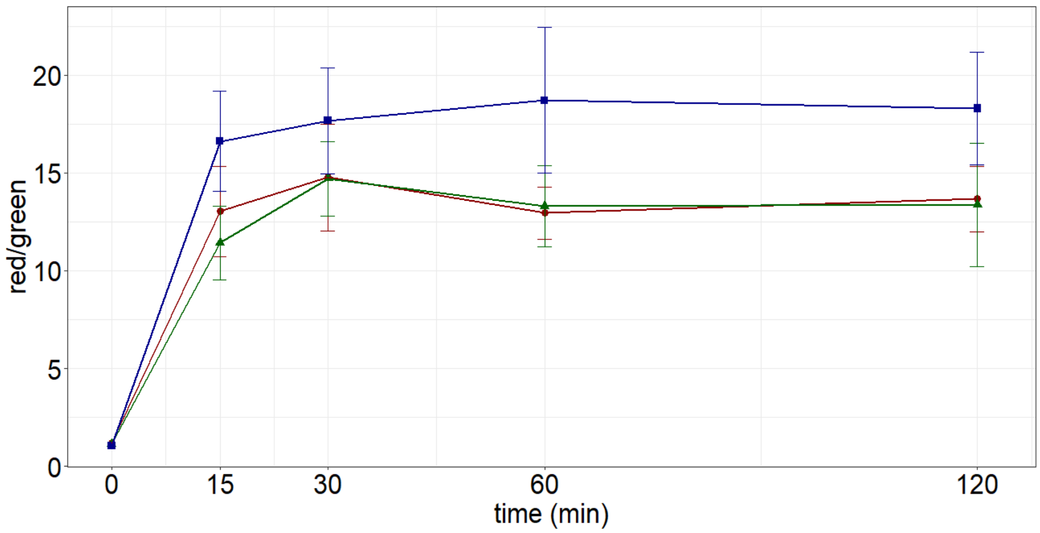

Figure SI2: Red-green-ratio of unexposed D. magna after different incubation times of JC-1 staining. Three independent experiments are shown. Error bars show standard deviation (n = 10) and no significant differences were measured between 15- and 120-minutes staining time (one-way ANOVA and Tuckey $p > 0.05$).

SI5 - OECD *D. magna* acute immobilization test for CCCP, 2,4-dinitrophenol, triclosan, 6PPD and ibuprofen

CCCP, 2,4-dinitrophenol, triclosan, 6PPD and ibuprofen were tested according to OECD guideline test 202 in 10 mL M7 in glass beakers. Immobilization was recorded after 2 h, 24 h and 48 h to determine a value that can be compared to the setup in 24-well plates. No mortality was observed in the control and DMSO control.

Table SI3. Dose-response curves and EC10 and EC50 values with corresponding 95 % confidence intervals of *D. magna* after 2 h and 24 h of exposure to CCCP, 2,4-dinitrophenol, triclosan, 6PPD, ibuprofen.

Chemical	Dose-response curves	EC_x	2 h	24 h	48 h
CCCP	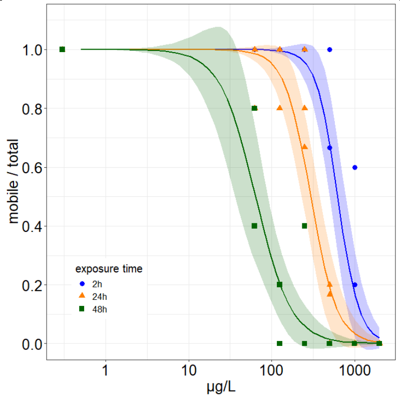	EC_{10} µg/L	625.2 (484.8 – 765.1)	296.1 (222.6 – 269.54)	63.9 (35.2 – 92.6)
		EC_{50} µg/L	327.3 (212.6 – 442.1)	133.5 (79.2 – 187.8)	21.6 (0.6 – 42.6)
2,4-dinitrophenol	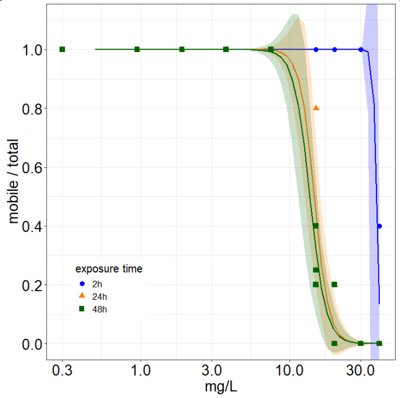	EC_{10} mg/L	14.6 (12.9 -16.4)	13.6 (11.6 – 15.7)	38.1 (30.1 - 46.1)
		EC_{50} mg/L	11.4 (8.42 -14.4)	10.4 (7.2 – 13.6)	35.9 (19.6 - 52.2)

triclosan	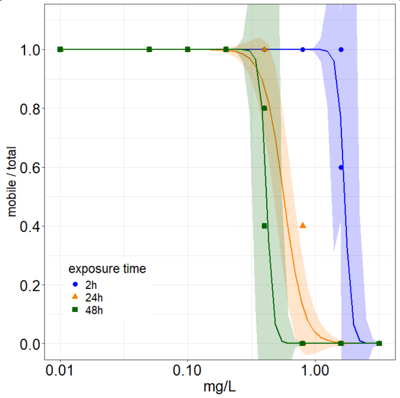	EC_{10} mg/L	1.70 (0.89 – 2.51)	0.57 (0.46-0.67)	0.42 (0.25 - 0.59)
		EC_{50} mg/L	1.49 (0.65 – 2.32)	0.38 (0.28 -0.49)	0.37 (0.04 -0.69)
6PPD	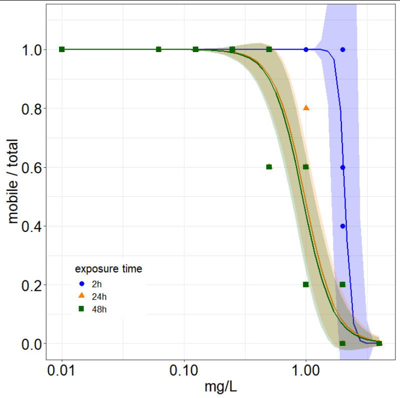	EC_{10} mg/L	2.09 (1.36 – 2.81)	0.98 (0.76 – 1.20)	0.93 (0.72 - 1.14)
		EC_{50} mg/L	1.82 (0.47 – 3.17)	0.52 (0.33 – 0.71)	0.50 (0.32 - 0.68)
ibuprofen	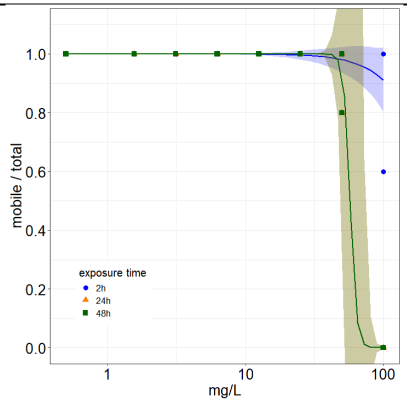	EC_{10} mg/L	No effect	57.35 (-4.39 -119.10)	57.35 (-4.39 -119.10)
		EC_{50} mg/L	No effect	51.16 (40.60 -61.72)	51.16 (40.60 -61.72)
